# Actin Up: An Overview of the Rac GEF Dock1/Dock180 and Its Role in Cytoskeleton Rearrangement

**DOI:** 10.3390/cells11223565

**Published:** 2022-11-11

**Authors:** Emily J. Koubek, Lorraine C. Santy

**Affiliations:** 1 Department of Neurology, University of Michigan, Ann Arbor, MI 48103, USA; 2 Department of Biochemistry and Molecular Biology, The Pennsylvania State University, University Park, PA 16802, USA

**Keywords:** Dock1, Dock180, Rac, Elmo, cytoskeleton

## Abstract

Dock1, originally Dock180, was the first identified member of the Dock family of GTPase Exchange Factors. Early biochemical and genetic studies of Dock180 elucidated the functions and regulation of Dock180 and informed our understanding of all Dock family members. Dock180 activates Rac to stimulate actin polymerization in response to signals initiated by a variety of receptors. Dock180 dependent Rac activation is essential for processes such as apoptotic cell engulfment, myoblast fusion, and cell migration during development and homeostasis. Inappropriate Dock180 activity has been implicated in cancer invasion and metastasis and in the uptake of bacterial pathogens. Here, we give an overview of the history and current understanding of the activity, regulation, and impacts of Dock180.

## 1. Introduction

Controlled re-organization of the actin cytoskeleton is critical for several important cellular processes, such as cell motility and invasion, clearance of apoptotic cells, myoblast fusion during muscle development, cell survival, and cell polarity. Rho GTPases (Rac, Cdc42, and Rho) are key players in the transduction of signals from the cell surface to the actin cytoskeleton. Of the Rho family of GTPases, Rac is known for its role in cell movement and induction of actin-rich lamellipodia protrusions at the leading edge. Small GTPases like Rac operate as “molecular switches” by flipping between active GTP-loaded and inactive GDP-loaded states. GTP-loading results in confirmational changes that allow Rac to bind specific effectors and initiate downstream signaling cascades. GTPase activation and inactivation is regulated by families of proteins that target the GTP-binding/GTP-hydrolytic cycle of GTPases. These include guanine nucleotide exchange factors (GEFs), and GTP hydrolysis-activating proteins (GAPs). On its own, GDP dissociates from small GTPases very slowly. GEFs promote GTP-GTPase binding by binding to GDP-bound GTPases and catalyzing the dissociation of GDP while simultaneously stabilizing the nucleotide-free GTPase. Since the cytosolic ratio of GTP is much higher than GDP, GTP then binds the empty GTPase. Binding of GTP to the GTPase subsequently release the GTPase-bound GEF, allowing it to activate another GDP-bound GTPase. GAPs, on the other hand, enhance the intrinsic GTPase activity of GTPases and promote GTPase hydrolysis of GTP to GDP. GDP then remains bound to the small GTPase until a GEF can catalyze its release. Thus, GEFs are considered small GTPase “activators” while GAPs are small GTPase “inhibitors” [1].

GEFs for Rho GTPases are subdivided into Dbl and DOCK (Dedicator of Cytokinesis) families. The DOCK family contains 11 mammalian members that play important roles in biological functions, development, and disease. In this review, we provide a comprehensive overview of the DOCK family member Dock180 (also known as Dock1). We discuss the biological role of Dock180 in Rac-induced signal transduction and cellular functions. Recent discoveries regarding the involvement of Dock180 in disease processes, such as cancer, are also highlighted.

## 2. Early Discovery

Dock180 was initially identified as a major binding partner of the adaptor protein Crk via Crk’s SH3 domain. Far Western blotting with the Crk SH3 domain identified binding of a cytosolic 180-kDa protein. Following molecular cloning of the identified cDNA, the protein was designated Dock180 (due to its 180-kDa size). Investigation of the amino acid sequence revealed a SH3 domain at the N-terminus and poly-pro Crk-binding sequences at the C-terminus. Presence of a SH3-binding domain, but absence of an SH2 domain, suggested that the protein associated with the cytoskeleton. Northern blotting detected Dock180 mRNA expression in placenta, lungs, kidneys, pancreas, and ovaries, but not peripheral blood leukocytes [2,3]. Membrane localized Dock180 promoted changes in 3T3 cell morphology from spindle-like to flat and polygonal, demonstrating that Dock180 can impact cytoskeletal structure and cell morphology [2,3].

Subsequent genetic studies identified orthologues of Dock180 in *Caenorhabditis elegans* and *Drosophila melanogaster*. Myoblast City (MBC) was first identified in a genetic screen and shown to disrupt myoblast fusion [4]. When the *mbc* gene was cloned, it was found to encode a homolog of Dock180 [5]. Both Dock180 and MBC contain the same three essential SH3 consensus residues in their SH3 domain and localize to the cytoplasm. MBC is expressed early in development and embryos carrying mutant *mbc* display defects in dorsal closure, cytoskeletal organization, and myoblast fusion. The *Drosophila rho*-like GTPase Drac1 is a homologue of the human small GTPase Rac1 and expression of *drac1* mutants resulted in abnormalities like those of mutant MBC. This suggested that MBC and Drac1 regulate myoblast fusion through the same pathway [5].

The *C. elegans* protein CED-5 was first identified in a screen for mutants that fail to remove apoptotic corpses and subsequent cloning found it to be highly homologous to Dock180 and MBC [6,7]. Interestingly, expression of Dock180 rescues mutant *ced-5* induced cell migration defects, indicating that the two proteins are functionally related [7]. Due to protein homology and functional similarities, CED-5, Dock180, and MBC were determined to be part of an evolutionarily conserved protein group, called the “CDM” family, which regulates cell migration, polarized cell surface extension, myoblast fusion, and apoptotic cell engulfment.

## 3. Identification of Rac Activating Activity

### 3.1. Dock180 in the Rac Signaling Pathway

As mentioned briefly above, the Rho family of small GTPases (Rac, Rho, and Cdc42) regulate cellular processes such as actin cytoskeleton rearrangements, focal adhesion signaling, cell migration and invasion, endocytosis, cell polarity, and axonal guidance. The role of each Rho-family member in a particular cellular pathway is tightly regulated in time and space by specific regulators and effectors. Detailed studies of the *mbc* mutant embryos found that in addition to disrupted myoblast fusion, these animals also showed impaired dorsal closure [5]. The similarities of these phenotypes to those seen in flies expressing dominant negative mutants of Rac lead these investigators to hypothesize that MBC played a role in Rac signal transduction [5]. *Drosophila* have a single receptor protein that is the fly homolog of the mammalian PDGF and VEGF receptors. This receptor (PVR) and its ligand PDGF/VEGF family 1 (PVF1) act as guidance receptors during the first phase of border cell migration during oogenesis in *Drosophila*. Expression of either mutant *mbc* or dominant negative Rac impairs border cell migration and attenuates the cell morphology disruption and actin accumulation induced by expression of an activated form of the PVR receptor, l-PVR [8]. Another screen in *Drosophila* designed to identify components of Rac signaling isolated multiple mutants in *mbc*, confirming its involvement with Rac regulation [9].

Multiple lines of evidence supported the idea that Dock180 regulates Rac activity in mammalian cells as well. Expression of either membrane-targeted Dock180 or constitutively active Rac promotes changes in cell morphology that are indistinguishable [10]. Expression of Dock180 induces JNK activation to the same extent as expression of constitutively active Rac and Cdc42 [9,10]. Additionally, several studies determined that overexpression of dominant negative Rac blocks Dock180-induced cellular changes, indicating that Dock180 is positioned upstream of Rac [9,10]. Based on these observations, Dock180 was identified as a key upstream regulator of Rac in Rac-mediated signaling.

Additional investigation of Rac signaling pathways found that integrin stimulation promoted formation of a complex containing Dock180 and the focal adhesion-associated proteins Crk and p130^Cas^. Formation of the complex induces cell spreading, phagosome formation, cell migration, and Rac activation [10,11,12,13]. Studies in *C. elegans* demonstrated that the worm versions of Crk (CED-2) and Dock180 (CED-5) interact and that they act upstream of Rac (CED-10) in cells that are engulfing apoptotic corpses [14]. Overexpression of dominant negative Rac, or Crk mutants unable to bind p130^Cas^ or Dock180, prevents engulfment and cell migration [12,13]. Additionally, expression of Dock180 leads to an increase in levels of GTP-bound Rac, but not GTP-bound RhoA or Cdc42, suggesting that Dock180 is a specific activator of Rac. Dock180-induced increases in GTP-bound Rac levels are further enhanced by co-expression of Crk and p130^Cas^ [10]. These observations strongly suggested that Dock180 acts in an integrin-stimulated signaling cascade and that recruitment of Dock180 to Crk/p180^Cas^ focal adhesion-localized complexes results in signal transduction from integrin to Rac activation.

### 3.2. Dock180 GEF Activity

As described above, members of the Rho family cycle between a GTP-bound “on” state and a GDP-bound “off” state. GEFs promote the exchange of GDP to GTP, thus “activating” Rho family members. Rho family GEFs include members of the classical Dbl-GEF family which have tandem Dbl-homology and Pleckstrin-homology (DH-PH) domains [15]. The DH domain contains the GEF catalytic activity, and the PH domain localizes the protein to the plasma membrane [15]. Originally, the mechanism by which Dock180 promoted Rac-GTP loading was unknown, and it was unclear if Dock180 is a Rac-GEF as it does not contain the Dbl-homology domain typical of other Rho-GEFs. A hallmark of any Rho-GEF is its ability to bind directly to a GTPase to stabilize the GTP- and GDP-free GTPase until binding of GTP and GEF dissociation. Indeed, Dock180 does physically interact with Rac, and not RhoA or Cdc42, in co-transfected cells [9,10]. Additionally, Dock180 appears to prefer binding of nucleotide-free Rac as Dock180/Rac binding increases in the presence of EDTA (which removes Mg^2+^ and increases GTP and GDP off rates), binding is lost in the presence of non-hydrolyzable GTP-γS- or GDP-loaded Rac1, and nucleotide-free Rac and Dock180 co-precipitate from cell lysate [9,10]. These data suggested that Dock180 may be a Rac-GEF.

**Figure 1:**
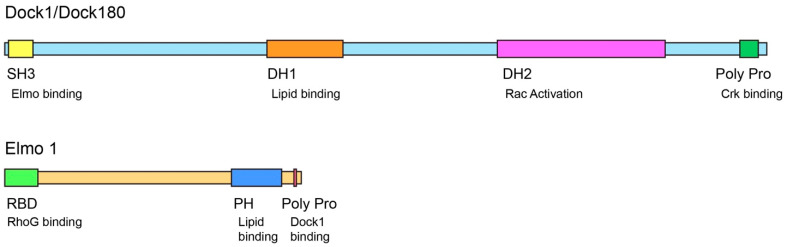
Domain Structure of Dock1/Dock180 and Elmo1. Dock180 is characterized by the evolutionarily conserved DH-1 domain that mediates PIP3 binding and the DH-2 domain that encompasses Rac GEF activity. The N-terminal SH3 domain mediates binding with the C-terminal poly pro domain of Elmo.

Dock180 contains a N-terminal SH3 domain and carboxy terminal proline-rich regions (Figure 1) [2,3]. BLAST analysis and pairwise alignment identified two additional protein domains, named DOCK Homology Region-1 and -2 (DH1 and 2). These regions contain high sequence homology and are evolutionarily conserved throughout the Dock180 superfamily (Figure 1) [16]. The DH1 domain is an unconventional phosphatidylinositol triphosphate (PtdInsP3)-interacting motif [17,18]. Interestingly, the DH2 region contains a tandem DH-PH domain that folds similarly to that found in other Rho family GEFs [16,19]. In vitro, the DH2 domain alone can bind nucleotide-free Rac and catalyze the exchange of GDP to GTP on purified Rac. Conversely, in vivo expression of a Dock180 mutant lacking the DH2 domain is unable to promote Rac-GTP loading [16,19]. The DH2 domain was specific for Rac as it did not bind to or promote GTP loading on Cdc42 or RhoA [16]. The DHR-2 domain is further organized into two regions: DH2N (amino acid residues 1178–1334) and DH2C (amino acid residues 1335–1657) [20]. The 300 residues at the C-terminal portion of the DH2 domain were shown to be necessary and sufficient for Rac GEF activity [20]. Additionally, the Ala^27^ and Trp^56^ of Rac uniquely interact with DH2C and act as a bipartite binding site for Dock180 [20]. This is in contrast with the Dbl GEF family that use Trp^56^ of Rac as the sole recognition site [20]. Several homologues and family members of Dock180 also contain the DH2 domain and bind and activate Rac1 and Cdc42 [16]. Dock180 was thus determined to be a novel Rac GEF that contains a newly identified conserved DH2 GEF domain.

## 4. Dock180 and Elmo: Better Together

### 4.1. Early Discovery of Elmo

To identify other proteins that may act with Dock180 and Crk to promote Rac activation, mutant embryos defective in distal tip cell (DTC) migration and cell corpse engulfment were screened in *C. elegans*. Embryos with mutant *Ced-12* displayed defects in DTC pathfinding and migration, removal of apoptotic cell corpses, and gonadal morphology [21,22,23]. These *Ced-12*-induced defects were phenotypically similar to those observed in mutants of the Crk, Dock180, and Rac *C. elegans* homologs *Ced-2*, *Ced-5*, and *Ced-10* and suggested that *Ced-12* may be a component of the same genetic signaling cascade [23]. This was further supported by the observation that co-expression of CED-12 and CED-5/Dock180 or CED-12 and CED-2/Crk enhanced phagocytosis while co-expression of all three proteins together displayed the highest engulfment efficiency [21]. Additionally, *Ced-2/CrkII*, *Ced-5/Dock180*, and *Ced-12* appeared to act at the same step in the pathway as overexpression of one gene did not compensate for the loss of any of the other two genes [22]. Downstream of Dock180 and Crk, overexpression of dominant negative CED-10/Rac blocked CED-12 induced phagocytosis and migration while wild type CED-10/Rac1 overexpression rescued *Ced-12* mutant-induced defects [21,22,23]. These results indicated that CED-12 acts in a signaling cascade at the same step as CED-2/Crk and CED-5/Dock180 upstream of CED-10/Rac during phagocytosis of apoptotic cells and cell migration in *C. elegans* [21,22,23].

Cloning of *Ced-12* and analysis of the protein sequence identified highly conserved orthologs in *Drosophila*, mice, and humans with the human ortholog identified as Elmo [21,22,23,24]. The Crk-Dock180-Elmo pathway is highly conserved and present in *Drosophila* and humans. In *Drosophila*, overexpression of MBC and Elmo together, but not alone, promotes myoblast fusion in the mesoderm and ommatidial organizational changes in the eye [24]. Additionally, MBC and Elmo co-expression prevents a dominant negative Rac-induced rough eye phenotype [24]. Overexpression of any Elmo homolog (Ced-12, Dced12, or Elmo) in murine Swiss 3T3 fibroblasts promotes similar actin remodeling [23]. In mammalian cells, overexpression of Elmo promotes actin polymerization. Co-expression of Crk, Dock180, and Elmo induces robust membrane ruffling and migration that is impaired by expression of dominant negative Rac [21,25]. Thus, Elmo is a conserved upstream regulator of Rac and acts in a Crk-Dock180-Elmo signaling pathway to promote cell migration and apoptotic cell engulfment.

As mentioned above, the observation that overexpression of one of the *Ced-2*/*CrkI*, *Ced-5*/*Dock180*, or *Ced-12/Elmo* genes does not compensate for the loss of the other two genes suggests that the three proteins act in the same step of the pathway and may physically interact to form a complex. CED-12/Elmo contains a PH domain and a complex proline-rich putative SH3 binding domain (Figure 1). Both domains are required for CED-12/Elmo function as mutants lacking the PH or SH3 binding domains do not rescue *Ced-12* mutant engulfment defects [23]. Under normal conditions, CED-12/Elmo localizes to the cytoplasm in a punctate pattern. However, CED-12 mutants lacking the PH domain display a diffuse, rather than punctate, cytoplasmic localization [23]. This suggests that the PH domain regulates localization of CED-12/Elmo to intracellular membranes [23]. In vitro binding assays indicated that the proline-rich region of CED-12/Elmo binds the N-terminal SH3 domain of CED-5/Dock180 [21,22,24,26,27]. In *C. elegans*, CED-12/Elmo binds CED-5/Dock180 and forms a complex with CED-2/Crk. Formation of the complex promotes recruitment to the plasma membrane, activation of CED-10/Rac, and cytoskeletal reorganization [22,23]. Similarly, *Drosophila* Elmo/CED-12 was isolated by MBC’s SH3 domain and stable MBC-Elmo complexes were identified during myoblast fusion by mass spectrometry [24]. In mammalian cells, both the C-terminal and N-terminal regions of Elmo are required for Dock180 binding and cell migration. Elmo co-precipitates with Dock180 via its C-terminal region while the N-terminal region of Elmo is required for localization of Elmo and Dock180 to lamellipodia and subsequent Rac activation and migration. Dock180-Elmo binding during migration is conserved as other members of the Dock180 super family, such as Dock2 and Dock3, also bind Elmo and promote mammalian cell migration when co-expressed with Elmo [25]. Interestingly, Elmo mutants lacking the first 531 amino acids bind Dock180 and promote Rac activation, however, they do not localize to membrane ruffles or induce mammalian cell migration. This suggests that during cell migration Rac must be activated by the Elmo-Dock180 complex at distinct intracellular locations and that localized activation of Rac is directed by the N-terminal region of Elmo [25]. Thus, Elmo not only promotes Rac activation, but also regulates Rac signaling. These observations suggested that Dock180-Elmo complex formation is required for Dock180 function.

### 4.2. Elmo and Dock180 GEF Activity: A Complicated Relationship

The mechanism of the Elmo/Dock180 interaction is complex and conflicting opinions existed regarding whether Elmo is required for Dock180 GEF activity. Early studies suggested that the interaction between Dock180 and Elmo is necessary for robust Rac GEF activity. This was based on the observation that expression of Elmo led to increased Dock180-nucleotide-free Rac binding and Rac-GTP levels and Dock180 deletions mutants unable to bind Elmo fail to activate Rac [19,25,26]. Additionally, formation of the Dock180-Elmo-Rac trimeric complex promotes Rac activation and subsequent Rac-induced phagocytosis and cell migration [26]. It was proposed that Elmo binds the Dock180 DH2 domain in trans and stabilizes the Dock180-Rac-Elmo trimolecular complex to increase affinity of Dock180 towards nucleotide-free Rac [26]. These studies led to the suggestion that Dock180 and Elmo1 are a 2-protein bipartite GEF [26,27]. However, other studies showed that expression of Dock180 or the DH2 GEF domain alone is sufficient to promote Rac GTP-loading, regardless of the presence of ELMO, calling into question the idea of an obligate bipartite GEF [10,16,28]. Mapping studies by Komander et al. identified hydrophobic residues on an N-terminal amphiphatic α-helix extension of the Elmo PH domain that directly bind to hydrophobic residues in an α -helical region flanking the SH3 domain of Dock180. A second interaction occurs between the C-terminal proline-rich motif (PXXP) of Elmo and the Dock180 SH3 domain [29]. Expression of an Elmo double mutant unable to bind Dock180 at either binding site did not affect Dock180-induced Rac activation. Similarly, a Dock180 double mutant incapable of binding Elmo was also able to promote Rac GTP loading. However, overexpression of the Elmo double mutant incapable of binding Dock180 does prevent cell spreading, elongation, and migration [29]. Thus, while Elmo-Dock180 complex formation is not required for Dock180 GEF activity, it is involved in Dock180-mediated Rac signaling.

**Figure 2:**
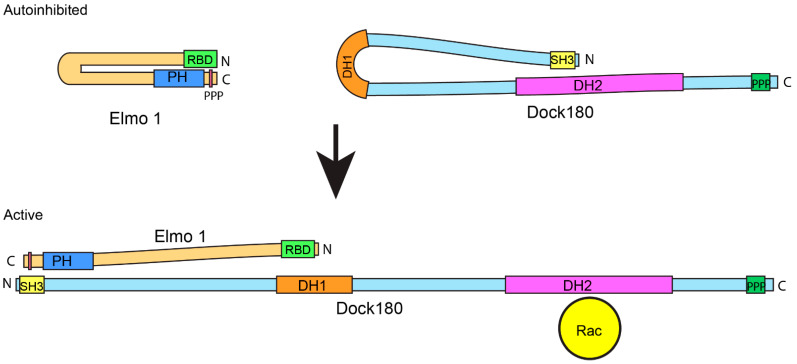
Control of Dock180 dependent Rac activation by autoinhibition. Both Dock180 and Elmo1 have intramolecular autoinhibitory interactions that control their activity. The N-terminal regions of Elmo bind to its C-terminus, while the Dock180 SH3 domain interacts with portions of the DH2 domain. When Elmo’s autoinhibitory interaction is disrupted then the Elmo poly-proline motif can bind to the Dock180 SH3 domain. This releases the DH2 domain which then binds to and activates Rac.

Recent work suggests that Elmo instead acts as a critical regulator of Dock180-mediated Rac signaling by relieving Dock180 autoinhibition and localizing the Elmo/Dock180 complex. Interestingly, Dock180 mutants lacking the SH3 region display enhanced phagocytosis, increased Rac1 binding, and increased Rac-GTP loading compared to wild type Dock180. This suggested that the SH3 domain may act as an intramolecular inhibitor of Dock180 GEF activity [27]. The SH3 region of Dock180 co-precipitates with the DH2 domain. Mutation of a conserved isoleucine residue (I32) within the SH3 region disrupts the SH3:DH2 interaction and enhances Dock180 phagocytic activity [27]. As the DH2 domain is responsible for Rac binding and GTP loading, the interaction between the N-terminal SH3 region and the catalytic DH2 domain sterically blocks Rac from interacting with Dock180. ELMO releases Dock180 autoinhibition by binding the SH3 region of Dock180 through its PXXP motif. Disruption of the SH3-DH2 interaction by Elmo binding allows Rac access to the DH2 domain and promotes Dock180 GEF activity [27] (Figure 2). Elmo itself also displays autoinhibition via intramolecular contacts that are released by protein binding. The N-terminal domain of Elmo known as the Elmo inhibitory domain (EID) contains Ras-binding domain (RBD) and Armadillo repeats which interact directly with the C-terminal Elmo autoregulatory domain (EAD) to prevent Elmo activity (Figure 2). Following engagement with Dock180, Elmo undergoes a confirmational change that releases its autoinhibition. Additionally, the active form of the Rho-family member, RhoG, interacts with the N-terminal RBD of Elmo and competes with the EID/EAD interaction. This appears to promote Elmo/Dock180 membrane recruitment and induce cell elongation [30].

## 5. Dock180 Signaling

### 5.1. Signal Transduction Leading to Dock180 Activation

**Figure 3:**
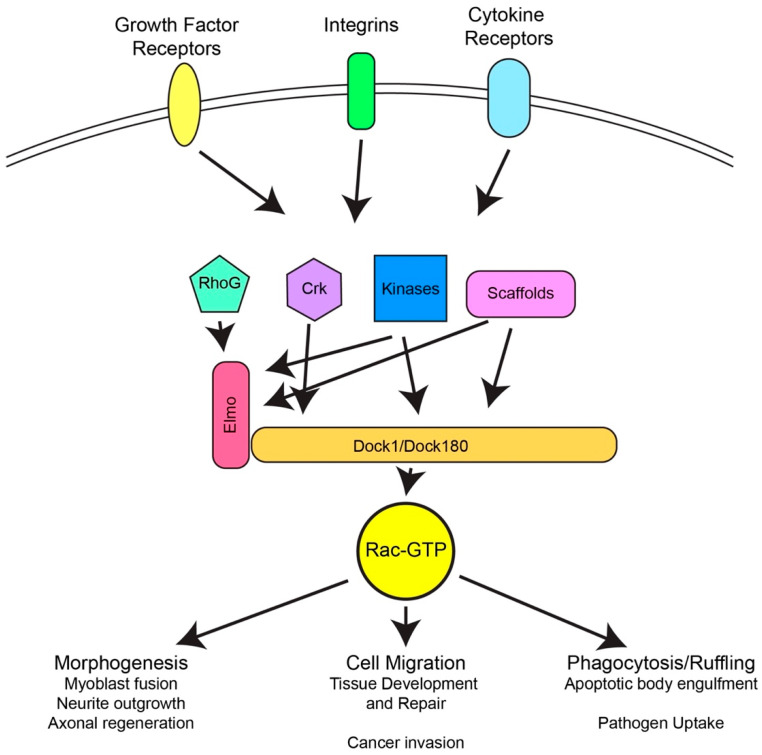
The Activators and Impacts of Dock180 dependent Rac Activation. Dock180 dependent Rac activation is enhanced by receptors via RhoG interaction with Elmo, Crk interaction with Dock180, phosphorylation of Elmo or Dock180 and/or scaffold protein dependent recruitment of Elmo or Dock180. The localized activation of Rac by Dock180 stimulates morphogenesis, cell migration and phagocytosis in a variety of normal and pathological contexts.

Upstream signaling to Dock180 leads to localized Dock180 dependent Rac activation, which participates in cell shape remodeling (Figure 3). As mentioned above, Dock180 was first identified as a binding partner for the adaptor protein Crk [2,3]. Crk is a proto-oncogene that binds to the tyrosine-phosphorylated tails of activated receptor tyrosine kinases and recruits downstream signaling molecules to these receptors. Thus, from its discovery, Dock180 was implicated in growth factor signaling and potentially in oncogenic transformation. These early studies also demonstrated that recruitment of Dock180 to the plasma membrane through artificial means led to altered cell morphology [2]. Subsequent studies determined that Dock180 is recruited to membranes and acts downstream of receptor tyrosine kinases [8,31,32,33,34,35,36,37], integrins [11,12], and chemokine receptors [38,39]. Both Crk-binding and phosphorylation of Dock180 by various kinases have been implicated in the activation of Dock180 in response to receptor signaling [11,12,33,34,36,40,41,42,43,44,45].

Additional signaling pathways can lead to the activation of Dock180 via the interaction of Elmo with active RhoG. Elmo was first identified as a binding partner for active RhoG through yeast 2 hybrid and biochemical assays [46]. RhoG dependent recruitment of the Elmo/Dock180 complex to the plasma membrane activates Rac in a Crk-independent manner, suggesting that RhoG mediates an alternate pathway for Dock180 activation. RhoG induced Dock180/Elmo dependent Rac activation promotes cell spreading and migration as well as the engulfment of apoptotic cells in both worms and mammalian cells [46,47,48].

Since Dock180′s original identification as a binding partner for the Crk scaffold protein, additional scaffolding proteins have been identified that bind to Dock180 or Elmo and recruit the complex to activate Rac during various cellular processes. These adapters include Nck-2 [49], Tamalin/GRASP [31,37,50], ANKRD28 [51], and FE65 [52]. Knockdown or mutation of these adaptors impairs Dock180-dependent cell migration and cell shape change.

In addition to protein–protein interactions, Dock180 is recruited to membrane surfaces by its lipid-binding DH1 domain [17,18,53,54]. The DH1 domain is a modified C2 lipid-binding domain that binds to PI-(4,5)P2 and PI-(3,4,5)P3 with similar affinity [54]. While lipid binding by Dock180 is not strictly necessary for Rac activation, it is required for proper localization of that activation and appropriate downstream signaling [17,18,54]. Therefore, Dock180 likely acts as a coincidence detector to integrate multiple upstream signals leading to a localized activation of Rac.

The standard model for localization of Dock180/Elmo is that the complex is directly recruited to its sites of action by binding to adapters and/or lipids. However, studies suggest that Dock180 interacts with vesicular trafficking systems and may be transported by these systems to its ultimate site of action. We found that ARF6 and the ARF-activating cytohesin proteins stimulate epithelial migration via Dock180-dependent activation of Rac [31,37,50,55,56]. The scaffold protein GRASP/Tamalin binds to both Dock180 and cytohesin-2, leading to their localization at recycling endosomes [31,37,50]. Signaling from the pro-migratory growth factor, HGF, leads to the transport of Dock180 from these endosomal compartments to the cell surface where it activates Rac [31,37]. More recently FE65 has been similarly shown to recruit Elmo to recycling endosomes and promote its subsequent movement to the plasma membrane [52]. Mutants of the *C. elegans* homologs of Dock180 and Elmo impair recycling of endocytic cargo in intestinal epithelial cells, suggesting that the Dock180/Elmo complex may be an active player in trafficking processes and not just cargo [57]. Finally, Dock180 has been reported to bind to sorting nexins and be required for proper transport of the cation-independent mannose 6-phosphate receptor [58].

In summary, signaling in response to growth factors, cytokines and integrins acts via kinases, lipid production, and scaffold proteins to produce localized activation of Rac by Dock180. These signaling proteins act to both relieve auto-inhibitory interactions in Dock180 and Elmo and to recruit them to regions of the plasma membrane. Dock180, Elmo and Rac are all peripheral membrane proteins that interact with the cytoplasmic leaflet of membranes. While they can be directly recruited from the cytoplasm to sites of action, the ability of vesicular trafficking processes to promote localized delivery of Dock180 and Elmo remains an understudied area of these signaling processes.

### 5.2. Impacts of Dock180 Activity

The result of signaling through Dock180 is localized activation of Rac and actin polymerization. The cytoskeletal alterations induced by Dock180 lead to changes in cell morphology and drive processes including migration, phagocytosis of apoptotic cells, myoblast fusion and neuronal development. Dock180-dependent Rac activation has also been implicated in cancer and infectious disease progression (Figure 3).

While Dock180 was first isolated by biochemical means as a binding partner of Crk [2,3], it was genetic studies in *Drosophila* with the Dock180 homolog MBC that first identified the consequences of Dock180 action and pointed to an interaction with Rac signaling in myoblast fusion and dorsal closure [5]. Further studies in *Drosophila* have cemented an essential role for Dock180 and Elmo in fly myoblast fusion [18,24]. Interestingly, these studies also suggest that interaction of Dock180 with Crk is dispensable for myoblast fusion [18]. Studies in vertebrates have confirmed a broadly conserved requirement for Dock180 function in muscle development. Knockout of Dock180 in mice [59] or its knockdown in zebrafish [60] impairs muscle development by blocking myoblast fusion. In contrast to the studies in flies, myoblast fusion in zebrafish requires Crk [60].

The involvement of Dock180 in the phagocytosis of apoptotic cell corpses was identified via genetic studies in *C. elegans* as already described above. Later studies on cell corpse engulfment identified CED-12/Elmo and demonstrated that it forms a complex with Crk and Dock180 to promote engulfment of cell corpses [21,22,61]. Further work has also found that the Crk/Dock180/Elmo complex is required for apoptotic cell clearance in mammals [11,21]. Interestingly, a recent study showed that, in addition to engulfment, Dock180 is able to directly interact with components of the autophagy pathway, such as LCR and Atg12, to regulate LC3-associated phagocytosis in mammalian cells [62].

The genetic screens that isolated MBC and CED-5, the fly and worm homologs of Dock180, were designed to identify genes involved in myoblast fusion and apoptotic cell clearance, respectively [4,5,6,7]. When these mutants were fully characterized, they provided in vivo evidence of Dock180′s involvement in the regulation of migration as well. Flies with mutations in *mbc* have defects in dorsal closure [5]. *Ced-5* mutant worms have misshapen gonads due to defective migration of the distal tip cells during gonad development [7]. Studies of the migration of border cells during *Drosophila* oogenesis identified MBC/Dock180 and Elmo as regulators of this directed migration process [8,32]. Mammalian cell culture studies using a variety of assays measuring migration and related processes confirmed that the ability of Dock180 and Elmo to regulate migration is conserved in vertebrates as well [25,31,40,46,56].

More recent studies have also demonstrated a role for Dock180/Elmo activity in neuronal development and regeneration. Increased plasma membrane recruitment of Elmo1 enhances neurite outgrowth in cultured primary rat cortical neurons [52,63]. Additionally, the RhoG/Elmo1/Dock180 signaling molecule is required for dendritic spine morphogenesis in cultured hippocampal neurons [64]. Finally, the CED-2/CrkII, CED-5/Dock180, CED-12/Elmo complex is required for axon regeneration after neuronal injury in *C. elegans* [65].

Additional studies have implicated Dock180/Elmo dependent Rac activation in other developmental processes as well. In embryonic stem cell derived embryoid bodies, Dock180 is essential to support the survival of the epiblast cells in contact with basement membrane, which go on to form a pseudostratified epithelial layer [41]. Mice expressing a mutant Dock180 that is unable to bind Elmo are embryonic lethal due to numerous cardiovascular abnormalities [39]. Studies in zebrafish support a role of Dock180/Elmo signaling in cardiovascular development. Elmo and Dock180 are expressed in the developing vasculature and knockdown of Elmo1 disrupts vascular development [66]. A follow up study found that Dock180 and Elmo1 protect endothelial cells from apoptosis, suggesting that they act as survival factors during angiogenesis [67].

## 6. Dock180 in Pathological Processes

### 6.1. Dock180 in Cancer

Early studies hypothesized that Dock180 would be involved in cancer progression given its ability to promote cell migration. Dock180′s involvement in cancer has been most extensively studied in glioma. Staining of human glioma samples found that Dock180 and Elmo were highly expressed in invasive glioma cells but not in the non-invasive body of the tumor [68]. They are also overexpressed in numerous glioma cell lines [68]. Knockdown of either Dock180 or Elmo in glioma lines impaired the ability of these cells to invade murine brain slices, while expression of Dock180 and Elmo in cell lines lacking them enhanced invasion [68]. Several growth factors have been shown to enhance migration and invasion of glioma cells via activation of Dock180/Elmo signaling [33,34,35,36]. Impairing the activation of Dock180 in glioma cells reduced the invasiveness of these cells both in vitro and in vivo [33,34,36]. Additionally, treatment of glioma cells with a small molecule that inhibits the interaction of Rac with Dock180 reduced glioma cell migration and invasion and enhanced glioma cell apoptosis [69].

Evidence linking Dock180/Elmo signaling with cancer progression is not limited to gliomas. Elmo1 is overexpressed in invasive ductal breast carcinomas compared to normal breast tissue [38]. Furthermore, knockdown of Elmo1 reduced lung metastasis but not primary tumor growth in a mouse breast cancer model [38]. Expression of Dock180 is elevated in ovarian carcinomas and high levels of Dock180 staining correlate with high tumor grade [70]. Like breast cancer and glioma cells, elevated expression of Dock180 in ovarian cancer cells enhances cell migration and invasion [23,38]. Dock180/Elmo signaling has therefore been implicated in invasion and metastasis in multiple types of cancer.

### 6.2. Dock180 in Infectious Disease

Dock180 was implicated in cellular invasion by the bacterial pathogens *Shigella* and *Campylobacter jejuni* in ways that mimic its role in apoptotic cell engulfment. These pathogens induce host intestinal epithelial cells to ruffle, which allows the pathogen to be surrounded by the host cell membrane and engulfed. *Shigella* injects a protein, IpgB1, into host cells that binds to Elmo and recruits the Elmo/Dock180 complex to the membrane surface to stimulate Rac activation and ruffling [71]. *Campylobacter* secretes extracellular matrix binding and membrane protein binding gene products that similarly stimulate Dock180-dependent Rac activation and ruffling [72,73]. This Dock180-induced ruffling and bacterial engulfment is similar to the Dock180-dependent membrane extension and apoptotic body engulfment that removes the remains of apoptotic cells.

In addition to involvement in cancer and infectious disease, mutations, or changes in Dock180 activity are likely to be involved in additional developmental defects and pathological processes. Whole genome sequencing and GWAS studies have identified alterations in the Dock180 gene in various pathologies, but the involvement of Dock180 in these processes remains to be confirmed, and mechanisms of action have yet to be investigated [74,75,76].

## 7. Conclusions and Perspectives

Here, we have provided an overview of the important role the Rac GEF Dock180 plays in several cellular functions, and also highlighted some of the recently elucidated involvement of Dock180 in pathological conditions. Dock180 is the canonical and original Dock protein, and studies of Dock180 have informed our understanding of the actions of all Dock family members. Dock180 and its partner Elmo are recruited by upstream signaling to activate Rac and stimulate actin polymerization at membrane surfaces. This activity drives cell shape changes during development, homeostasis, and pathological processes.

The physiological roles that have been investigated for Dock180 dependent Rac activation were generally identified by phenotypes found in model organism mutants. While this has been a fruitful approach to this point, a comprehensive understanding of Dock180 in development and homeostasis will require a systematic approach to studying the role of this protein in a variety of tissues. These studies will require the use of tissue-specific mouse knockout models since mice lacking Dock180 are not viable [59]. It is likely that Dock180 plays an essential, yet unappreciated, role in additional developmental cell migration and tissue morphogenic processes. Receptor induced migration is often reactivated after development during tissue repair. The role of Dock180 during this essential process remains to be investigated. We have found that cytohesin-stimulated Rac activation, which requires Dock180 in vitro, is essential for HGF-stimulated repair after ischemic kidney injury [31,77]. It would be interesting to investigate the involvement of Dock180 in the recovery of additional tissues where reactivation of migration has been implicated in tissue repair [78]. Similarly, a comprehensive understanding of the involvement of Dock180 in cancer progression will require systematic investigation of changes in Dock180 sequence and expression levels in a variety of cancers. Such studies are likely to identify additional conditions that depend upon the function of this, no longer unconventional, GEF.
